# Unpredictable Nanoparticle Retention in Commonly Used Plastic Syringes Introduces Dosage Uncertainties That May Compromise the Accuracy of Nanomedicine and Nanotoxicology Studies

**DOI:** 10.3389/fphar.2019.01293

**Published:** 2019-11-06

**Authors:** Uwe Holzwarth, Unai Cossío, Jordi Llop, Wolfgang G. Kreyling

**Affiliations:** ^1^Joint Research Centre (JRC), European Commission, Ispra, Italy; ^2^Radiochemistry and Nuclear Imaging, CIC biomaGUNE, San Sebastian, Spain; ^3^Centro de Investigación Biomédica en Red, Enfermedades Respiratorias–CIBERES Parque Tecnológico de San Sebastián, San Sebastián, Spain; ^4^Institute of Epidemiology, and Institute of Lung Biology and Disease, Helmholtz Zentrum München–German Research Center for Environmental Health, Munich, Germany

**Keywords:** nanoparticle retention in plastic syringes, disposable plastic syringes, dosage uncertainty, dosage reproducibility, *in vivo* studies, nanomedicine, nanotoxicology

## Abstract

In recent animal experiments with suspensions of radiolabeled TiO_2_ nanoparticles large and highly variable radioactivity fractions were retained in disposable plastic syringes. After unloading between 10% and up to 70% of the loaded dose were still present in the syringes. As a consequence the effectively delivered nanoparticle dose to the animals was frequently much smaller than the nominal dose of the nanoparticles loaded into the syringe. The high variability of this nanoparticle retention challenges the application of a precise, predefined dose and creates a major error source when normalizing organ and tissue contents to the dose loaded into the syringe, which is usually set as the applied dose. A control study was performed employing six commonly used syringe types with seven types of radiolabeled oxide and metallic nanoparticles. For this purpose the syringes were loaded with a given volume of nanoparticle suspension, the radioactivity was measured, the syringe was unloaded and the activity measurement was repeated with the empty syringe. The highest retention values were found when using TiO_2_ nanoparticle suspensions with Tuberkulin type syringes. In the worst case between 6.6% and 79.1% of the nanoparticles were retained in the syringe. When using the same nanoparticle suspension with an insulin-type syringe the retention was reduced to 1.4% to 20.6%. For amorphous silica nanoparticles the maximum observed retention was 8% and for Au nanoparticles it was 5.1%. Further data gathered from *in vivo* animal imaging studies show that nanoparticle retention in syringes also affects experiments with nanoparticles such as exosomes, polymersomes, and protein-based nanoparticles investigated for possible applications in nanomedicine. Since the retention is highly variable the effectively applied dose cannot be determined by applying a simple syringe retention factor. The present work shall alert to the problem and illustrate its possible magnitude and unpredictable variability. As mitigation strategy adequate checks with different syringe types are proposed in order to find out whether a given combination of syringe type and nanoparticle suspension is affected by nanoparticle retention and, if necessary, to select a different syringe type that minimizes retention.

## Introduction

*In vivo* experiments with nanoparticles for biodistribution or biokinetics studies are frequently carried out with disposable plastic syringes to apply a certain volume of a nanoparticle suspension intravenously (Kreyling et al., 2017a). This is also common practice for oral administration *via* a stomach catheter or for various forms of lung application (Kreyling et al., 2017b; Kreyling et al., 2017c). *In praxi* it is usually assumed that after emptying the syringe all nanoparticles that were loaded into the syringe with a given volume of a stock suspension are also administered to the animals, thus knowing precisely the initial quantity and concentration.

In a series of *in vivo* experiments with radiolabeled [^48^V]TiO_2_ nanoparticles executed at the Helmholtz Zentrum Munich completely balanced biodistribution studies were performed including all tissues, organs, and all collected excretions of the animals (Kreyling et al., 2017a; Kreyling et al., 2017b; Kreyling et al., 2017c). In these experiments the [^48^V]TiO_2_ nanoparticles amounts were quantified by γ-ray spectrometry making use of the γ-ray emissions of the radiolabel ^48^V. With this very reliable and sensitive method discrepancies between the radioactivity balance of the animal specimens and the quantity of nanoparticles loaded into the syringe for application were noted. On the other hand the unloaded, i.e., seemingly empty syringes exhibited unexpected high radioactivity doses when they were checked for radioactive waste disposal although dead-spaces in the syringes were carefully avoided as described by Kreyling et al. (2017a; 2017b; 2017c). The detected amounts of nanoparticles retained in the syringes were in the range of about 10% to nearly 70% of the loaded dose. The retentions matched very well with the amounts that were missing in the activity balances obtained in the biodistribution studies (Kreyling et al., 2017a; Kreyling et al., 2017b; Kreyling et al., 2017c). Knowing the effectively applied dose from the animal’s radioactivity balances, it was still possible to derive valid biokinetics and biodistribution data in organs and tissues. However, in view of the huge scatter of syringe retention, experiments applying the same dose to each animal were not possible. If organ fractions were conventionally normalized to the dose loaded into the syringe, as it is common practice in *in vivo* studies, a syringe retention between 10% and 70% would lead to normalized *in vivo* organ contents that are underestimated between a factor of 1.1 and 3.3 hampering the statistical analysis between different animals.

A literature research revealed only one publication by Keene et al. (2012) that mentioned dosage uncertainties with nanoparticles in animal experiments. These authors found that up to 20% of their gold nanoparticles remained in the syringe/needle complex after injection. To the authors’ knowledge, this is the only report of this kind in nanobiosciences literature. However, Keene et al. (2012) attributed their finding to nanoparticle agglomeration, not considering the possible role of the syringe type and without further reflecting on dosage accuracy and reproducibility.

Extending the literature research beyond nanoparticle applications revealed that syringe retention of certain types of ^99m^Tc-labeled compounds is known in nuclear medicine since it may compromise imaging quality (Gunasekera et al., 2001; Gmeiner Stopar et al., 2007; Swanson et al., 2013; Bauwens et al., 2014; Galbraith et al., 2015; Rahman and Galbraith, 2015; Reynolds and Kikut, 2015; Taillefer, 2016). In some cases retention in syringes was even imaged (Swanson et al., 2013; Bauwens et al., 2014). In nuclear medicine the proper choice of the syringe for a medical imaging compound is recommended to be part of the ongoing quality control procedures (Gmeiner Stopar et al., 2007; Swanson et al., 2013; Bauwens et al., 2014; Reynolds and Kikut, 2015; Taillefer, 2016).

The experimental findings and the experience gathered in nuclear medicine triggered a series of syringe retention experiments with radiolabeled nanoparticles executed at the European Commission’s Joint Research Centre at Ispra (Italy), shortly named JRC. These simple experiments were performed with the only scope to quantify the retention of nanoparticles in different types of commonly used disposable plastic syringes in order to broaden the data base of unexpected syringe retention after unloading. Since the JRC activities focus on the safety of industrially applied nanoparticles it was important to complement the analysis with nanoparticles that are considered for applications in nanomedicine. For this purpose a large amount of syringe retention data from *in vivo* animal imaging studies with radiolabeled nanoparticles were re-examined. These studies were performed during recent years at the Center for Cooperative Research in Biosciences in San Sebastian (Spain), shortly named CIC biomaGUNE in the following.

Investigations of the physicochemical reasons for the highly variable interaction of nanoparticles with syringes is beyond the scope of the present work due to the plethora of parameters resulting from the numerous syringe types and the huge number of physicochemical properties of the many different nanoparticle suspensions. However, even without a satisfactory solution of the issue immediate action is required in view of the resource intensive efforts to reduce experimental scatter in nanobiosciences by providing well characterized representative nanomaterials out of industrial production (Totaro et al., 2016) and reference materials to reduce scatter caused by calibration uncertainties (Stefaniak et al., 2012; Roebben et al., 2013;Roebben et al., 2015). These efforts must not be compromised by avoidable dosage inaccuracies.

## Materials and Methods

### Experiments Performed At the JRC

#### Materials

In a first step the same type of pure anatase nanoparticles of the type ST-01 from Ishihara Ltd (Japan) that were used in the *in vivo* studies by Kreyling et al. (2017a; 2017b; 2017c) were ^44^Ti diffusion labeled. Two further TiO_2_ nanomaterials taken from the JRC Nanomaterials Repository (Rasmussen et al., 2014) were diffusion labeled with ^44^Ti, the P25 from Evonik (Germany) and the material NM-104 which had a thin Al_2_O_3_ surface coating. All diffusion-labeled nanoparticles were suspended in sterile ultrapure water without dispersants. These materials [^44^Ti]TiO_2_ ST-01, [^44^Ti]TiO_2_ P25, and [^44^Ti]TiO_2_ NM-104 were tested with three different syringe types.

Diffusion-labeling was performed by wetting 10 to 20 mg of nanoparticles with [^44^Ti]TiCl_4_ solution (purchased from Los Alamos National Laboratory, Los Alamos, New Mexico 87545, USA) in a glass vial and subsequent heat treatment at 180°C for 150 min in a vacuum furnace at slightly reduced ambient pressure (850 mbar). The dry nanoparticle powder was recovered in 20 ml sterile, ultrapure MilliQ water in a glass vial and subjected to ultrasound homogenization for 45 min using a Branson Digital Sonifier 450 equipped with a tapered microtip applying an energy dose of about 400 J/ml. The temperature increase during ultrasound treatment was limited to (25–30°C) by immersing the glass vial in a 500 ml ethanol-ice bath. By centrifugal filtration of 0.5 ml suspension using a 30 kDa filter the residual amount of free ^44^Ti was determined to be below 0.1% indicating a labeling yield above 99.9%. The very low values of free ^44^Ti could be confirmed up to 8 weeks after radiolabeling showing that the ^44^Ti-radiolabels were stably integrated in the nanoparticles.

The same procedure was applied to label the amorphous silica nanoparticles NM200 (Rasmussen et al., 2013) taken from the JRC Nanoparticle Repository. Centrifugal filtration as described above revealed nearly two thirds of the used ^44^Ti in the filtrate. Therefore the suspension was washed using a 10 kDa centrifugal filter twice with 2 ml MilliQ water, 12 times with 2 ml 0.25 M HCl and another five times with 2 ml MilliQ water which resulted finally in less than 0.01% free ^44^Ti and allowed to determine the radiolabeling yield as 37%. When repeating the washing on 0.5 ml of aqueous stock suspension 4 weeks after radiolabeling the fraction of free ^44^Ti in the filtrate was as low as 0.03% which indicates stable integration of the ^44^Ti radiolabel in the [^44^Ti]SiO_2_ nanoparticles.

The [^48^V]TiO_2_ E171 food additive was radiolabeled by proton irradiation (executed by the Zyklotron AG, Karlsruhe, Germany), which has already been described in much detail in literature (Holzwarth et al., 2012) applying the same method as in our *in vivo* studies (Kreyling et al., 2017a; Kreyling et al., 2017b; Kreyling et al., 2017c).

Citrate stabilized aqueous suspensions of [^195^Au]Au nanoparticles and of [^195^Au]Au/[^110m^Ag]Ag core/shell nanoparticles were synthesized from radioactive precursors ([^195^Au]HAuCl_4_ and [^110m^Ag]AgNO_3_). The gold cores were synthesized by adding 1 ml of 25 mM [^195^Au]Au^3+^ solution to 150 ml 2.2 mM sodium citrate solution at 97°C for 20 min. After cooling down to room temperature a size selection and washing procedure was applied by centrifugation to select a core size of about 8 nm. 2 ml of these seeds, readjusted to a concentration of 0.25 mM, where then added to 0.85 ml of 11 mM [^110m^Ag]AgNO_3_, 46 ml H_2_O, and 1.25 ml 0.1 M sodium citrate and maintained at a temperature of 80°C for 20 min.

#### Methods

At the JRC six different types of 1 ml syringes have been used for the experiments in combination with seven types of radiolabeled nanoparticles which had been labeled for various purposes. The materials the syringes were made of (according to the manufacturers) are reported in **Table 2** together with the nanoparticle retention results. For three types of syringes only 10 individual syringes were available. Therefore they could not be tested with all nanoparticle suspensions as we wanted to use only syringes of a certain brand and type that were from the same lot.

The standard procedure to determine the syringe retention of these nanoparticles was as follows: In order to exclude dead volume effects, before loading the syringes with nanoparticle suspensions, the plunger was put in position 0.1 ml which created an air volume in the syringe barrel that was greater than the highest specified dead volume, in order to ensure that all liquid was pushed out when unloading the syringe. Of course this method cannot be applied when intravenous administration to animals would be foreseen.

Before filling any syringe the stock solutions were vortexed for 1 min. Then the syringes were loaded with 0.5 ml of aqueous nanoparticle suspension and the activity was determined by γ-ray spectrometry using a High Purity Germanium detector properly calibrated in energy and efficiency. For this purpose a multiline γ-ray source was used that contained ^241^Am, ^109^Cd, ^60^Co, and ^137^Cs to cover also γ-ray energies below 100 keV (^241^Am: 59.5 keV; ^109^Cd: 88 keV) required for the reliable quantification of ^44^Ti and ^195^Au. The calibration was controlled using a certified ^152^Eu reference source with known activity whose γ-ray emissions cover the energy range from 121.7 to 1,408 keV and with additional ^241^Am and ^109^Cd calibration sources. The radioactivity of the loaded syringes was measured for 20 min, then they were immediately unloaded, and the radioactivity of the unloaded syringe was re-measured using the same detector and the same measurement geometry. In order to minimize hypothetical effects that might be caused by the speed of pushing the plunger all experiments were performed by the same operator emptying the syringe within about 5 s. Retention was determined as the activity ratio of the activity determined for the unloaded syringe divided by the value determined for the loaded syringe. Due to the short time delay of about 20 min between the two measurements it was not necessary to correct the activities for decay when calculating the ratio (physical half-lives ^44^Ti:*T*_1/2_ = 60.4 y; ^48^V: *T*_1/2_ = 15.97 d, ^195^Au *T*_1/2_ = 186.1 d; ^110m^Ag: *T*_1/2_ = 248.8 d).

The ^44^Ti was quantified using its γ-ray emissions at 67.9 and 78.3 keV. The ^48^V-labeled material was quantified by its main γ-ray emissions at 983.5 and 1,312.1 keV. The [^195^Au]Au nanoparticles were quantified by the 98.9 keV γ-rays emitted by the ^195^Au. Also the [^195^Au]Au/[^110m^Ag]Ag core/shell nanoparticles were quantified by the γ-rays emitted from the ^195^Au in the nanoparticle core in order to exclude any misinterpretation by ionic ^110m^Ag that might accompany the suspension due to a slow dissolution of the Ag shell.

### Data Compiled and Evaluated at CIC biomaGUNE

CIC biomaGUNE has a long record of *in vivo* studies with radiolabeled nanoparticles for medical purposes applying also nuclear animal imaging techniques. The nanoparticles were synthesized in-house or were provided by collaboration partners for radiolabeling, in order to assess their suitability for applications in nanomedicine. In these studies the effectively applied dose was always determined from the difference of the radioactivity loaded into the syringe minus the activity retained in the unloaded syringe. While in this way the applied dose was always precisely known problems with the reproducible application of a precisely prescribed dose were frequently noted.

For the present study no new experiments were performed because the existing data pool contained all the relevant information, which was extracted and evaluated in order to provide the fraction of activity, i.e., nanoparticles that were retained in the unloaded, seemingly empty syringes.

#### Materials

Information concerning the synthesis, functionalization, and characterization of the more than 40 types of nanoparticles can be found in the literature cited in **Table 3** (Conde-Vancells et al., 2008; Locatelli et al., 2012; Pérez-Campaña et al., 2012; Pérez-Campaña et al., 2013; Frigell et al., 2014; Locatelli et al., 2014; Pérez-Campaña, 2014; Ruggiero et al., 2015; Gil, 2016; Ruggiero et al., 2016; Pulagam et al., 2017; Simón-Gracia et al., 2018; Royo et al., 2019). Here we have to limit ourselves to details which have not yet been published and are complementary to the cited literature.

*Preparation of [* *^68^* *Ga]GNRs-1@PNPs-NODA*. A suspension (5 ml, 0.14 mM) of gold nanorods (GNRs) coated with ethyl 11-mercaptoundecanoate (**1**), which were entrapped into polymeric nanoparticles (PNPs). (GNRs-**1**@PNPs) was prepared in PBS (20 ml, 0.01 M) as described by Locatelli et al. (2014). To this suspension a solution of *N*-hydroxysulfosuccinimide (23 mM, 1.74 ml) as well as a solution of 1-ethyl-3-(3-dimethylaminopropyl) carbodiimide (2.8 M, 0.71 ml; previously adjusted to pH 7.5 with diluted HCl) were added under permanent stirring. The solution was left to react at room temperature for 30 min then 2.5 mg (3.61 µmol) of NODA-GA-ethylenediamine dissolved in 1 ml of water (previously adjusted to pH 7.5 with diluted NaOH) was added, and the system was left to react overnight. The mixture was then concentrated and purified by washing with PBS (three times) using centrifugal filter devices (Amicon Ultra, Ultra cell membrane with 100.000 NMWL, Millipore, USA), to a final volume of 5 ml. Finally GNRs-1@PNPs-NODA were filtered using syringe filters (phenex-PES of polyether sulfone; 26 mm, 0.20 μm, Phenomenex, Italy). Labeling with ^68^Ga was performed by incubation (*T* = 60°C; *t* = 15 min) of the NPs with ^68^GaCl_3_ using 4-(2-hydroxyethyl)-1-piperazineethanesulfonic acid (HEPES) as buffer (final pH = 3.80 ± 0.05). The ^68^Ga-labeled GNRs-**1**@PNPs-NODA were filtered under centrifugation (10,000 rpm, 5 min) using Amicon^®^ Ultra-4 Centrifugal Filter Units (50 kDa, Millipore Corporation). The residues were washed twice with HEPES solution (pH = 3.65) and finally suspended in physiologic saline solution for administration into animals.

*Preparation of* *^18^* *F-Labeled Upconverting Nanoparticles ([* *^18^* *F]UCNP)*. The synthesis of NaGdF_4_:Yb^3+^/Er^3+^ (rare earth element ratio 78/20/2 in mol%) upconverting nanoparticles (UCNPs) was carried out following a procedure customized for NaYF_4_ nanoparticles and previously published (Ruggiero et al., 2015; Ruggiero et al., 2016). The functionalization of UCNPs with phosphonate ligands (alendronic acid, citric acid, or nitrilotri(methylphosphonic acid) to yield UCNP-3P, UCNP-CITRIC and UCNP-ALE, respectively) was carried out as follows: 30 mg of NaGdF_4_:Yb^3+^/Er^3+^ UCNPs were dissolved in 2 ml of chloroform, and 100 mg of the phosphonate ligand were dissolved in 10 ml of 2-(*N*-morpholino)ethanesulfonic acid (MES) buffer (20 mM). The solution mixture was then stirred overnight, and the aqueous phase (supernatant) lyophilized. The obtained solid material was washed three times in ethanol (3 ml) to eliminate the excess ligand and then left for drying at room temperature. Radiofluorination was achieved by incubation of the NPs with [^18^F]F^−^ in aqueous media, followed by purification using centrifugal filtration.

*Preparation of [* *^124^* *I]MLP29 Exosomes*. The exosomes generated from Mouse Liver Progenitor (MLP)-29 cells were prepared as previously reported by Conde-Vancells et al. (2008). For the radiolabeling, solutions of the exosomes (20 µg/20 μl) were incubated with Na[^124^I]I (37 MBq) in PBS (20 µL, 0.5 M, pH = 7.4) for 2 h at 25°C in an Iodination Tube (Royo et al., 2019). Gentle periodic shaking was applied. When the reaction was finished, the crude was diluted with PBS solution (250 µL, 0.01 M, containing NaCl 1 M, pH = 7.4) and the resulting solution was transferred to a vial containing Na_2_S_2_O_3_ (50 µL, 0.1 M). Finally, the resulting solution was purified on a Sephadex™ Column using PBS (0.01 M, containing NaCl 1 M, pH = 7.4) as the mobile phase. The eluted product was collected in 100 µL fractions and those containing the highest concentration of radioactivity were diluted with physical saline solution and used for *in vivo* experiments.

*Preparation of [* *^124^* *I]Au and Ag Nanoparticles*. Gold and silver nanoparticles were synthesized and functionalized by direct methods from HAuCl_4_ and AgNO_3_ using appropriate thiolate ligands. Two different methods were followed for the preparation of gold nanoparticles by reduction of Au^3+^ to Au^0^: the Turkevich method and a modified Brust and Schiffrin method. Silver nanoparticles were synthesized by reduction of Ag^+^ to Ag^0^ with NaBH_4_. Both gold and silver nanoparticles were first purified by precipitation with an organic solvent and then by dialysis in pure water over a 5-day period. The Ag and Au nanoparticles were characterized locally by UV-vis and FTIR​. Before radiolabeling the NPs were sonicated in ultrasound bath for 5 min. The NPs were then incubated with Na[^124^I]I. Purification of the labeled NPs was carried out by centrifugal filtration using Millipore filters (3 kDa cutoff, Amicon Ultra-0.5 ml). Three consecutive washing steps were performed before final resuspension to conduct *in vivo* experiments.

#### Methods

CIC biomaGUNE determined the applied nanoparticle dose from the difference of the radioactivity loaded into the syringe minus the activity retained in the unloaded syringe after administration. The radioactivity measurements were performed in a dose calibrator (CRC-25R dose calibrator, Capintec Inc, Ramsey, NJ, USA) calibrated following the procedure established by the manufacturer (available on line at: https://capintec.com/support/manuals/crc-25r-dose-calibrator-manual/ ; Chapter 7: Acceptance & quality assurance tests).

## Results

The results of the syringe retention experiments performed at the JRC are compiled in **Table 2**. When using the same type of syringe (brand A/2) and the same type of nanoparticles (TiO_2_ ST-01 (Ishihara Ltd, Japan)) which were used in the *in vivo* experiments using [^48^V]TiO_2_ ST-01 nanoparticles (Kreyling et al., 2017a; Kreyling et al., 2017b; Kreyling et al., 2017c) we obtained in five repetitions with [^44^Ti]TiO_2_ nanoparticles retentions between 16.6% and 63.5% which matched the range and variability observed earlier (Kreyling et al., 2017a). In the worst case using the syringe type Brand B/1 between 6.6% and 79.1% of the [^44^Ti]TiO_2_ nanoparticles of the type ST-01 were retained, while using the same nanoparticle suspension with an Insulin-type syringe (Brand A/3) reduced the retention to 1.4% to 20.6%.

For amorphous silica nanoparticles the maximum observed retention was 8%, for Au/Ag core/shell nanoparticles, the maximum retention was 6.4% and for Au nanoparticles it was 5.1%. Thus, the experiments performed at the JRC exhibit low syringe retention for metallic nanoparticles and amorphous silica nanoparticles, and it might be concluded that that mainly TiO_2_ nanoparticles are affected by syringe retention. However, the data compiled in **Table 3** on [^13^N]Al_2_O_3_ nanoparticles by Pérez-Campaña et al. (2013) show another type of metal oxide nanoparticle which exhibits syringe retentions that may exceed peak values of 70%. Moreover, the data provided in **Table 3** on functionalized Au nanoparticles show that [^124^I]Au functionalized with –NH groups exhibit a retention around 2%, close to the value in **Table 2** when using the same syringe type (Brand A/3) with non-functionalized Au nanoparticles, but when using a different functionalization such as [^124^I]Au-Cosan-PEG retention may reach about 50%.

**Table 3** compiles syringe retention data of more than 40 types of nanoparticles (core material and functionalization) of medical interest that were radiolabeled and tested *in vivo* at CIC biomaGUNE at concentrations relevant for medical applications (Conde-Vancells et al., 2008; Locatelli et al., 2012; Pérez-Campaña et al., 2012; Pérez-Campaña et al., 2013; Frigell et al., 2014; Locatelli et al., 2014; Pérez-Campaña, 2014; Ruggiero et al., 2015; Gil, 2016; Ruggiero et al., 2016; Pulagam et al., 2017; Simón-Gracia et al., 2018). The syringes used in these experiments were of the type brand A/3 and brand A/1 which were among those tested at the JRC (see **Table 1**). From **Table 3** it becomes evident that syringe retention is a phenomenon that affects also polymeric and protein-based nanoparticles and exosomes and appears to be of a general nature.

**Table 1:** Main characteristics of the nanoparticles used by the JRC in the present investigation.

	DLS/*z*-average	DLS/PDI	ζ-Potential	Primary particle size	Crystalline structure	Concentration
[^44^Ti]TiO_2_ ST01	(170.1 ± 1.1) nm	0.134 ± 0.007	(41.6 ± 1.3) mV	8 nm (XRD)	Anatase	250 µg/ml
[^44^Ti]TiO_2_ P25	(148.4 ± 15.2) nm	0.215 ± 0.034	(24.6 ± 0.8) mV	22 nm anatase (XRD)/48 nm rutile (XRD)	Anatase ≈ 85%/Rutile ≈15%	500 µg/ml
[^44^Ti]TiO_2_ NM01004a	(127.5 ± 1.5) nm	0.212 ± 0.009	(27.3 ± 1.1) mV	(26 ± 10) nm (TEM)	rutile	400 µg/ml
[^48^V]TiO_2_ E171	(356.2 ± 8.3) nm	0.213 ± 0.028	– (9.0 ± 0.4) mV	(137 ± 112) nm (TEM)	Anatase ≈ 98%	300 µg/ml
[^44^Ti]SiO_2_ NM-200	(211.0 ± 1.5) nm	0.266 ± 0.010	– (30.7 ± 0.4) mV	(18 ± 5) nm (TEM)	Amorphous	40 µg/ml
[^195^Au]AuNP 15nm	(14.6 ± 0.6) nm	0.467 ± 0.040	– (39.9 ± 5.4) mV	(8.2 ± 1.4) nm (TEM)	Face centered cubic	33 µg/ml
[^195^Au]Au/[^110m^Ag] Ag Core/shell NPs	(19.4 ± 0.6) nm	0.228 ± 0.009	– (41.1 ± 0.6) mV	(16 ± 4) nm (TEM)	Face centered cubic	10 µg/ml

All nanoparticles were used in aqueous suspension in MilliQ water at pH ≈ 7 and in concentrations that are typical for in vivo experiments. The ^44^Ti diffusion labeled TiO_2_ materials were ST-01 (Ishihara Ltd, Japan), Aeroxide® P25 (Evonik, Germany), and two materials from the JRC Nanomaterials Repository, NM01004a TiO2 (earlier named NM-104) (Rasmussen et al., 2014), which had a thin alumina coating, and amorphous SiO_2_ type NM-200 (Rasmussen et al., 2013). Characterization was performed with dynamic light scattering (DLS); the z-average and the polydispersity index (PDI) were determined. Since DLS determines the hydrodynamic particle size, nanoparticle size was determined either by X-ray diffraction (XRD) or transmission electron microscopy (TEM).

The data in **Table 2** using nanoparticle suspension in at least three and up to six types of syringes reveal that the retention depends on the nanoparticle-syringe combinations used in these experiments. However, the most concerning feature of the syringe retention effect is that it exhibits a high and unpredictable variability even when using the same combination of syringe type of nanoparticle suspension.

**Table 2:** Summary of the JRC syringe retention measurements; The number n of syringes is presented, the mean retention value ± its standard deviation in percent and the range from the lowest to the highest determined retention value.

	Brand A/1 Tuberkulin Luer tip, 1ml	Brand B/1 Tuberkulin Luer tip, 1ml	Brand C/2 Luer tip, 1ml with needle	Brand A/2 Tuberkulin Luer tip, 1 ml	Brand C/1 U-100 Insulin 0.33 × 12.7 mm	Brand A/3 U-100 Insulin0.33 × 12.7 mm
Needle	Braun Sterican, 22G x 1¼”				Stainless Steel 304	Stainless Steel
Material barrel	Polypropylene	Polypropylene	Polycarbonate	Polypropylene	Polypropylene	Polypropylene
Material plunger (rod)	Polyethylene	Polyethylene	Polypropylene	Polystyrene	Polystyrene	Polystyrene
Material plunger seal	n.a.	n.a.	Latex-free elastomer	Polyisoprene	Polyisoprene	Polyisoprene
Lubricant	Silicone-free	Silicone-free	Med. grade silicone	Silicone	Silicone	Silicone
[^44^Ti]TiO_2_ ST01	**n = 16**	**n = 9**	**n = 5**	**n = 5**	**n = 11**	n = 5
	**(31.6 ± 23.0) %**	**(28.6 ± 26.7) %**	**(23.8 ± 22.2) %**	**(37.2 ± 22.8) %**	**(9.2 ± 8.3) %**	(10.3 ± 7.8) %
	**3.2% – 74.1%**	**6.6% – 79.1%**	**5.4% – 51.9%**	**16.6% – 63.5%**	**0.7% – 27.6%**	1.4% – 20.6%
[^44^Ti]TiO_2_ P25	n = 6	n = 6			n = 6	
	(14.6 ± 2.9) %	(7.7 ± 1.8) %			(3.9 ± 1.7) %	
	9.2% – 17.2%	5.6% – 11.0%			2.2% – 7.0%	
[^44^Ti]TiO_2_	**n = 7**	n = 7			n = 4	
NM01004a	**(26.2 ± 8.6) %**	(11.9 ± 5.9) %			(9.6 ± 5.3) %	
	**15.3% – 39.9%**	6.1% – 20.3%			2.6% – 14.2%	
[^48^V]TiO_2_ E171	**n = 6**	**n = 6**			n = 3	n = 3
	**(25.6 ± 14.3) %**	**(14.9 ± 16.1) %**			(8.2 ± 6.3) %	(6.2 ± 6.2) %
	**12.4% – 46.1%**	**2.2% – 45.4%**			4.0% – 15.4%	1.1% – 13.1%
[^44^Ti]SiO_2_ NM-200	n = 7	n = 5		n = 2		
	(7.3 ± 3.8) %	(4.5 ± 2.0) %		(4.5 ± 2.0) %		
	1.6% – 13.8%	3.2% – 8.0%		3.0% – 5.9%		
[^195^Au]AuNP 15nm	n = 5	n = 6	n = 5	n = 3	n = 5	n = 2
	(2.5 ± 0.9) %	(2.0 ± 1.3) %	(2.0 ± 0.6) %	(3.9 ± 1.1) %	(1.9 ± 1.1) %	(1.1 ± 1.1) %
	1.3% – 3.5%	1.1% – 4.5%	1.6% – 3.0%	3.1% – 5.1%	1.0% – 3.6%	0.3% – 1.9%
[^195^Au]Au/[^110m^Ag]AgCore/shell NPs	n = 3	n = 3			n = 3	
	(3.8 ± 0.6) %	(4.8 ± 1.8) %			(1.1 ± 0.5)%	
	3.1% – 4.3%	2.9% – 6.4%			0.5% – 1.5%	

Cases in which syringe retentions higher than 25% have been determined are highlighted in boldface. The syringe materials and properties are given as far as available from the manufacturer documents.

## Discussion

The investigation of the interaction of nanomaterials with biological systems appears to be especially affected by a lack of reproducibility and comparability of data (Baker, 2016; Faria et al., 2018). This triggered a lot of efforts to harmonize the execution and reporting on experimental studies and to improve the reproducibility, credibility, and efficiency of scientific research. These efforts comprise the set-up of repositories of well-characterized representative industrially manufactured nanomaterials (Totaro et al., 2016), the development of reference nanomaterials (Roebben et al., 2015; Roebben et al., 2013) and the recommendation of Minimum Information Reporting in Bio-Nano Experimental Literature (MIRIBEL) (Faria et al., 2018). Unfortunately, these efforts are compromised by the unexpected retention of nanoparticles in disposable plastic syringes used for *in vivo* experiments.

The data compiled in **Tables 2** and **3** illustrate the magnitude and high variability of nanoparticle retention in plastic syringes. The high variability, for which we have no reasonable explanation, is the most concerning feature because it makes it impossible to compensate the problem by applying a simple numerical syringe retention factor. It causes a dosage uncertainty with unpleasant repercussions on all experimental data and statistical analysis which are elaborated by normalization to a nominally applied dose, which is usually set to the nanoparticle dose loaded into the syringe. The lack of reporting in nanobiosciences literature concerning differences between a nominally and an effectively applied dose and the related checks sheds doubt on the accuracy and reproducibility of the results.

**Table 3:** Summary of syringe retention and nanoparticle characterization data obtained at CIC biomaGUNE: The number of syringes (n), mean retention and standard deviation and range are given.

Type of nanoparticles	Citation	DLS/*z*-average (nm)	DLS/PDI	ζ-pot. (mV)	TEM primary particle size (nm)	Conc. (mg/ml)	Brand A/1Tuberkulin Luer tip, 1 ml	Brand A/3U-100 Insulin 0.33 × 12.7 mm
**Nonfunctionalized metal oxide NPs**	****	****	****	****	****	****	****	****
[^18^F]Al_2_O_3_	Pérez-Campaña et al., 2012; Pérez-Campaña, 2014	8.4 ± 1.9			5.3 ± 1.2	1.6	n = 8**;(21.14 ± 7.30)% 12.10%-30.00%**	
[^13^N]Al_2_O_3_- NS_10nm_	Pérez-Campaña et al., 2013	26.7 ± 4.9		3.2 ± 0.4	12.2 ± 4.1	30	**n = 6;(19.94 ± 5.14)% 12.70%-28.37%**	
[^13^N]Al_2_O_3_- NS_40nm_		33.1 ± 8.1		18.0 ± 3.3	31.0 ± 19.0	30	**n = 6;(27.17 ± 8.50)% 17.54%-38.36%**	
[^13^N]Al_2_O_3_- NS_150nm_		271.3 ± 18.9		10.6 ± 2.8	178 ± 68	30	**n = 6;(56.85 ± 18.67)% 31.58%-73.99%**	
[^13^N]Al_2_O_3_- NS_10µm_		2,351 ± 109		–5.8 ± 1.6		30	**n = 9;(59.53 ± 12.23)% 33.33%-73.02%**	
**Functionalized metallic nanoparticles**	****	****	****	****	****	****	****	****
[^68^Ga]C_11_GNP	Frigell et al., 2014				2.4 ± 0.2	0.2	n = 2;(18.91 ± 6.63)% 14.21%-23.60%	
[^68^Ga]LipGNP					2.2 ± 0.2	0.2	n = 2;(19.69 ± 1.93)% 18.33%-21.05%	
[^68^Ga]Lip-Enk GNP					2.1 ± 0.2	0.2	**n = 3;(21.20 ± 11.03)% 11.78%-33.33%**	
[^68^Ga]Lip-glycopep GNP					2.2 ± 0.2	0.2	n = 2;(18.88 ± 0.01)% 18.87%-18.88%	
[^68^Ga]C_11_-glycopep GNP					3.2 ± 0.3	0.2	**n = 3;(34.99 ± 6.03)% 29.96%-41.67%**	
[^68^Ga]C_11_-Enk GNP					2.7 ± 0.3	0.2	**n = 3;(25.38 ± 13.94)% 10.50%-38.13%**	
[^124^I]Ag COOH		47 ± 1	0.5	–27 ± 1		0.42		**n = 1;45.45%**
[^124^I]Ag NH		109 ± 10	0.3	49 ± 5		0.42		n = 1;2.04%
[^124^I]Ag PEG		28 ± 1	0.4	–23 ± 6		0.42		n = 1;4.55%
[^124^I]Au_5_-NH		444 ± 135	0.6	26 ± 3		0.42		n = 2;(1.89 ± 0.67)% 1.42%-2.37%
[^124^I]Au_5_-PEG		359 ± 77	0.5	–32 ± 4		0.42		n = 2;(16.99 ± 0.61)% 16.55%-17.42%
[^124^I]Au_20_-PEG		211 ± 27	0.4	–29 ± 1		0.83		n = 2;(6.19 ± 0.89)% 5.56%-6.82%
[^124^I]Au Cosan PEG	Pulagam et al., 2017	20.8 ± 0.5		–18.0 ± 0.7	19.2 ± 1.4	1.0		**n = 5;(41.08 ± 9.51)% 28.87%-50.27%**
**Polymeric nanoparticles**	****	****	****	****	****	****	****	****
[^67^Ga]PMAAc-NH_2_-NODA-GA	Gil, 2016	35	0.3			0.25		n = 5;(16.81 ± 5.33)% 11.46%-24.02%
[^67^Ga]PMAAc-NH_2_-NODA-GA-PTR_86_-FITC		38	0.4			0.25		n = 5;(17.97 ± 3.38)% 12.41%-21.30%
[^67^Ga]PMAAc-NH_2_-NODA-GA-tPA		37	0.3			0.25		n = 5;(13.71 ± 3.60)% 9.55%-18.60%
[^67^Ga]PMAAc-NH_2_-NODA-GA-PTR_86_-FITC-PEG-MMP-substrate-PEG		52	0.6			0.25		**n = 5;(17.74 ± 13.22)% 8.67%-40.86%**
[^67^Ga]PMAAc-NH_2_-NODA-GA-tPA-PEG-MMP-substrate-PEG		39	0.4			0.25		n = 5;(16.90 ± 4.61)% 9.14%-21.37%
[^67^Ga]PMAAc-NH_2_-NODA-GA-PTR_58_-FITC		40	0.45			0.25		**n = 5;(18.92 ± 7.13)% 11.22%-29.70%**
[^67^Ga]PMAAc-NH_2_-NODA-GA-PTR_58_-FITC-PEG-MMP-substrate-PEG		46	0.68			0.25		n = 5;(5.29 ± 1.98)% 3.40%-7.68%
[^67^Ga]PEG-PLGA-Fe_3_O_4_-NODA		154 ± 2.0	0.28	– 10.5		0.25		n = 2;(7.43 ± 3.47)% 4.98%-9.88%
[^67^Ga]PEG-PLGA-Fe_3_O_4_-NODA-PTR_86_-FITC		90.0 ± 1.8	0.24	– 38.2		0.25		n = 2;(7.78 ± 0.26)% 7.60%-7.96%
[^67^Ga]PMAAc-NH_2_-DOTA-GA		75	0.3			0.25		n = 2;(12.78 ± 4.34)% 9.71%-15.84%
[^67^Ga]PMAAc-NH_2_-DOTA-GA-PTR_86_-FITC		148	0.4			0.25		n = 2;(15.64 ± 8.56)% 9.59%-21.70%
[^67^Ga]PAMAM_inj_-CAN-Fe_3_O_4_		34.0		53.5		0.25		**n = 4;(22.46 ± 7.02)% 13.20%-29.99%**
[^67^Ga]PAMAM_inj_-CAN-Fe_3_O_4_-PTR_86_-FITC		144.5		43.8		0.25		**n = 4;(46.70 ± 36.32)% 12.24%-79.17%**
[^68^Ga]Magh-1-PNPs-NODA	Locatelli et al., 2012	92.34 ± 0.72	0.167± 0.011	–44		0.012*		n = 2;(4.55 ± 0.14)% 4.45%-4.65%
[^68^Ga]GNRs-1@PNPs-NODA^[b]^	Locatelli et al., 2014	147.8 ± 0.81	0.24	–60.1				**n = 19;(26.97 ± 7.41)% 17.54%-45.08%**
[^124^I]LinTT1-Tyr-PS	Simón-Gracia et al., 2018	124 ± 62	0.15	–0.1 ± 4.0	44.4 ± 9.99	10		n = 5;(3.19 ± 2.34)% 0.99%-6.33%
[^124^I]Tyr-PS		138 ± 65	0.22	3.1 ± 3.3	55.6 ± 10.0	10		n = 4;(3.88 ± 3.00)% 1.01%-6.49%
**Protein-based NPs**	****	****	****	****	****	****	****	****
[^67^Ga]CAN-Fe_3_O_4_-rHSA-NOTA-NHS	Gil, 2016	120 ± 1.4				0.25		n = 4;(14.22 ± 5.82)% 6.29%-19.84%
[^67^Ga]CAN-Fe_3_O_4_-rHSA-NOTA-NHS-PTR_58_-FITC						0.25		**n = 5;(24.47 ± 15.18)% 8.40%-41.04%**
[^67^Ga]CAN-Fe_3_O_4_-rHSA-DOTA-NHS		174 ± 6	0.11	16.5 ± 2.0		0.25		n = 6;(13.82 ± 2.33)% 10.18%-16.74%
[^67^Ga]CAN-Fe_3_O_4_-rHSA-NOTA-NHS-PTR_86_-FITC		164 ± 4	0.09	17.1 ± 2.2		0.25		n = 6;(9.11 ± 2.34)% 5.80%-11.81%
[^67^Ga]CAN-Fe_3_O_4_-rHSA-NOTA-NHS-tPA		181 ± 14	0.13	21.4 ± 1.5		0.25		**n = 4;(21.18 ± 3.91)% 17.11%-26.53%**
**Functionalized nanocrystals**	****	****	****	****	****	****	****	****
[^18^F]UCNP-3P	Ruggiero et al., 2015; Ruggiero et al., 2016	1,404	1		9.5 ± 1.1		**n = 3;(32.36 ± 11.73)% 18.82%-39.60%**	**n = 1;22.08%**
[^18^F]UCNP-CITRIC					9.5 ± 1.1		**n = 2;(41.61 ± 2.58)% 39.78%-43.43%**	****
[^18^F]UCNP-ALE		805.1	0.91		9.5 ± 1.1		**n = 3;(23.49 ± 7.52)% 14.81%-28.02%**	**n = 4;(47.34 ± 22.56)% 25.00%-74.03%**
Exosomes								
[^124^I]MLP29 Exosomes	Conde-Vancells et al., 2008			40.6 ± 11.2				**n = 9;(16.22 ± 7.93)% 10.34%-36.11%**

*Concentration of iron in the injected solution. The hydrodynamic particle size was determined as the z-average by dynamic light scattering (DLS) in many cases the primary particle size was determined by transmission electron microscopy (TEM), as far as available the polydispersity index (PDI) and the ζ-potential are presented The mass concentration of the nanoparticles used in the experiments is given. The synthesis is described in the cited literature. Cases in which syringe retentions higher than 25% have been determined are highlighted in boldface.CAN, cerium ammonium nitrate; DOTA, 1,4,7-tetraazacyclododecane-1,4,7,10-tetraacetic acid; Enk, enkephalin; FITC, fluorescein isothiocyanate; GNP, glucose coated gold nanoparticles; GNRs, gold nanorods; Lip, lipoic acid; Magh, maghemite nanoparticles; NH2-NODA-GA, 2,2,’-(7-4(-((2-aminoethyl)amino)-1-carboxy-4-oxobutyl)-1,4,7-triazonane-1,4-diyl)diacetic acid; NS, nominal size; PAMAM, polyamidoamide dendrimer; PEG, polyethylene glycol; PLGA, Poly(D,L-lactide-co-glycolide); PMMAc, polymethacrylic acid; PNPs, polymeric nanoparticles, polyethyleneglycol-based nanoparticles; PTR, peptidic somatostatin analog conjugate; rHSA, recombinant human serum albumin; tPA, tissue plasminogen activator peptide ligand.

According to the knowledge of the authors the only mentioning of nanoparticle retention in syringes is a statement made by Keene et al. (2012) that up to 20% of Au nanoparticles were not injected in their laboratory animals. However, this was probably attributed too hastily to the effect of agglomeration of nanoparticles which was the main subject of their study. This might explain why their ‘alert’ has not been recognized as such in a broader context. However, nanoparticle aggregation was meticulously controlled in the studies of Kreyling et al. (2017a; 2017b;2017c) where utmost care was taken to control particle size before application to a group of animals.

A look at the retention values compiled in **Tables 2** and **3** underlines the quantitative similarity with retention phenomena encountered in nuclear medicine. For example, Galbraith et al. (2015) showed that “non-reactive” syringes, (i.e. in their terminology those that were not lubricated with silicon oil) retained (6.38 ± 2.95)% of ^99m^Tc-succimer while silicon oil containing syringes retained (30.6 ±12.5)%. The individual values ranged from 1.5% to 17.4% and from 8.3% to 73.9%, respectively. However, a look at the syringe properties included in **Table 2** shows that the presence or not of silicon oil cannot explain the present findings. The adsorption of various ^99m^Tc-labeled compounds, such as ^99m^Tc-sestamibi and ^99m^Tc-succimer to syringes is considered a significant problem in nuclear medicine both in terms of magnitude and due to its unpredictable variability (Swanson et al., 2013; Galbraith et al., 2015; Taillefer, 2016). In nuclear medicine retention values up to about 10% to 15% (Gmeiner Stopar et al., 2007) of loaded activity are considered acceptable. In **Tables 2** and **3** we applied a more generous criterion and highlighted (in boldface) those cases where 25% or higher nanoparticle retention was seen in single experiments.

The similarity of the results compiled in **Tables 2** and **3** shows in addition that the retention phenomenon is not affected by the methods of radiolabeling. The retention of chemically labeled nanoparticles where the radiolabel is hold by chemical bonds—usually attached on the nanoparticle surface *via* a functional linker molecule—shows the same features that are observed for oxide nanoparticles which are physically radiolabeled by direct proton beam exposure (Holzwarth et al., 2012) or by a diffusion treatment (Hildebrand et al., 2015). Both physical methods will distribute the radiolabels in the whole nanoparticle volume without a preference to locations on or close to the surface (Butz, 2012; Holzwarth et al., 2012; Hildebrand et al., 2015). Considering also the high stability of the radiolabeled constructs, the frequently large percentages of radioactivity retained in syringes cannot be attributed to any type of a hypothetical exchange effect of the radiolabel between the nanoparticles and the plastic materials of the syringes. This would also not explain the high variability of the results. Furthermore, any type of radiation-mediated effect between the syringe materials and the nanoparticles can be excluded since the activities injected into animals for imaging are nearly three orders of magnitude higher (MBq vs kBq range) than those prepared for *in vitro* and *in vivo* studies at the JRC and the Helmholtz Zentrum Munich, respectively. In this case we would expect a systematic difference in the data between **Table 2** and **3**. This is not observed. Furthermore, many plastic syringes are sterilized applying radiation doses which are again several orders of magnitude higher than those they may have received from their radiolabeled load (Karpuz and Özer, 2016).

The activity measurements of the full and the empty syringe reported in the present work were performed immediately before and after unloading the syringe. Therefore, even some release of ^124^I from polymersomes during several hours (Simón-Gracia et al., 2018), a maximum 10% release of the radiolabel after 72 h from exosomes (Royo et al., 2019) or of < 5% of ^68^Ga from biodegradable polymeric nanoparticles in 8 h (Locatelli et al., 2012) will not compromise the results. Moreover, if we assume that the label will be released in ionic form it will be eliminated from the syringe during unloading with the liquid phase. Thus, if the syringe retention results will be affected at all, they will be underestimated.

Intuitively, we tried to explain the findings by attractive electrostatic effects between the syringe plastic materials and the nanoparticles and tried to find a relation between syringe retention and the ζ-potential data of the nanoparticles reported in **Tables 1** and **3**. However, when pooling the data no convincing relation could be identified. It appears that neither the polarity nor the magnitude of the ζ-potential have any predictive power for nanoparticle retention. Especially it cannot explain its high variability. If we consider the ζ-potential as a nanoparticle property, which is characteristic for a given nanoparticle suspension, how can the same ζ-potential explain that for a certain combination of syringe type and nanoparticle suspension the retention values can cover a range from, e.g., 10% to nearly 80%? All arguments indicate that the syringes should be examined more thoroughly considering that electrostatic charges may be created by plastic components sliding against each other (Galembeck et al., 2014). However, as long as a physicochemical reason of this effect has not been unambiguously identified, the only feasible mitigation strategy appears to be the experimental identification of the brand and type of syringe that allows the most reproducible dosage of a given nanoparticle in a given suspension as recommended in nuclear medicine by including such checks in the ongoing quality control procedures (Gmeiner Stopar et al., 2007; Swanson et al., 2013; Bauwens et al., 2014; Reynolds and Kikut, 2015; Taillefer, 2016).

In **Tables 2** and **3** we anonymized the syringe brands since we want to avoid the wrong impression of giving any recommendations. Since the type of syringe that worked well for [^44^Ti]TiO_2_ ST-01 (**Table 2**) did badly for example with [^67^Ga]PAMAM_inj_-CAN-Fe_3_O_4_-PTR86 (**Table 3**), the performance of syringes cannot be generalized, and it has been pointed out that the findings might already be different after the manufacturer decided to modify or update a seemingly uncritical step in the manufacturing process of the syringe (Swanson et al., 2013). Therefore, syringe performance may even depend on the lot number (Swanson et al., 2013) and it appears necessary prior to each experimental series to perform the checks again whenever materials or processes were modified or changed.

It is obvious that the development of medicines is too expensive to accept early pre-clinical *in vivo* studies being compromised by artifacts that may be avoided by somehow cumbersome but comparably inexpensive precautions of adequate quality control. Rigorous reporting of the results of such checks should be promoted to increase knowledge on and awareness of such retention effects. In a recent meritorious work Faria et al. (2018) promote a minimum information standard for experimental work investigating bio–nano interactions Minimum Information Reporting in Bio-Nano Experimental Literature (MIRIBEL, cf. https://doi.org/10.17605/OSF.IO/SMVTF) in order to counteract the ‘reproducibility crisis’ (Baker, 2016) that jeopardizes scientific credibility, and compromises progress in nanomedicine and regulatory decisions on the safe use of nanomaterials. Unfortunately MIRIBEL is incomplete concerning the assessment of accuracy and reproducibility of dosage in the light of the present findings, and an amendment appears necessary.

The reader may conclude that we advocate the use of radiotracers, but this is not necessary to analyze and manage syringe retention problems. For example, whenever mass spectroscopic methods can be applied to quantify nanoparticles, it will be possible to determine syringe retention by flushing the unloaded syringe with the acids used to digest the nanoparticles for analysis before syringe disposal. Each laboratory should develop its method based on the quantification method it is familiar with in order to implement adequate precautions.

The question why certain substances interact with syringe materials in such a pronounced and unpredictable way should be answered by materials scientists and with the help of medical device producers. In the introduction of the ISO Standard 7886-1/2017 it is acknowledged that it is not possible to check each injection solution with all available syringes. However, this ISO standard refers to the possible interaction effect of solvents in the solutions with the syringe materials. In the present case we have not a contamination problem. Standards dealing with the fabrication of medical devices (such as ISO 7886-1, 2017) as well as those for biocompatibility testing (ISO 10993-1, 2003) focus on the risk of contamination by substances released from the material the device is made of. ISO 8537, 2016 on “Sterile single use syringes, with or without needle, for insulin” specifies only that “materials used (…) shall not (…) adversely affect the efficacy, safety, and acceptability of insulin preparations. The fabrication materials shall also not be affected (…) by insulin preparations.” The standard does not specify any specific material choice. However, we observed that insulin syringes avoid the use of polyethylene and we could find published evidence for the interaction of insulin with polyethylene (Cecil and Robinson, 1975). Thus, we believe that a special choice of materials and may be changes in manufacturing procedures should be envisaged for syringes dedicated to nanoparticle applications.

For *in vitro* testing researchers can resort to a vast offer of “non-binding” lab-ware, where “non-binding” refers to proteins. This experimental requirement was satisfied by manufacturers developing certain surface modifications of their lab-ware. Nevertheless, *in vitro* testing during pharmaceutical development still faces problems with lab-ware binding to the substance under investigation which may cause significant differences between the nominal concentration and the bioavailable concentration of a substance (Groothuis et al., 2015; Kramer et al., 2015; Vinken and Blaauboer, 2017). This entails the risk to underestimate the toxicity of a substance by *in vitro* testing (Vinken and Blaauboer, 2017). Besides the data presented here nothing similar is reported so far for *in vitro* testing of nanoparticles. However, a recent report dealing with the quantification of nanoparticle surface hydrophobicity shows that surfaces can be manipulated in a way to completely bind or repel nanoparticles (Valsesia et al., 2018). This means that binding problems may indeed occur during *in vitro* testing with nanoparticles, but it seems encouraging that “non-binding” lab-ware and syringes could become available if surface modifications can be implemented in an industrial production process of medical devices. For the time being, if both the *in vivo* dose as well as the *in vitro* dose may be affected by undesired interaction of the nanoparticles with syringes and lab-ware, respectively, utmost care has to be taken to check for retention effects in each experiment.

For *in vivo* experiments such considerations are *de facto* a legal requirement since the Directive 2010/63/(EU, 2010) on the protection of animals used for scientific purposes explicitly asks for an “experimental or observational strategy and statistical design to minimize animal numbers.” Therefore, steps to increase awareness on syringe retention and to implement appropriate measures are mandatory in order to ensure that the replacement–reduction–refinement of animal use is adequately implemented (Daneshian et al., 2015;Graham and Prescott, 2015).

## Conclusions

The presented data show that for certain comEUbinations of a nanoparticle suspension and syringe type an accurate and reproducible dosage is not possible since a highly variable fraction of the nanoparticles is retained in the unloaded syringe after administration.

According to the authors’ knowledge control experiments to check for a possible retention of nanoparticles in syringes have never been reported so far in literature. Therefore, we conclude that this issue has been overlooked or at least been underestimated so far. We assume that syringe retention of nanoparticles may be one of the reasons for the scatter of nanotoxicological data in literature. In the development of nanomedicines this effect could obscure the reproducibility of pre-clinical testing of nanomedicines if different syringe types are used which may hamper and delay progress in pharmacological development.

The data indicate that using a different type of syringe may reduce the problem to acceptably low and less variable values. This finding is in line with the results reported in nuclear medicine for certain ^99m^Tc-labeled imaging compounds. As long as it is not possible to rely on the suitability of a syringe for the precise *in vivo* administration of a given nanoparticle dose in bio-nanosciences the same mitigation strategy should be applied as recommended in nuclear medicine. This means that the retention of nanoparticles in the syringe should be quantified before starting with *in vivo* experiments and if necessary other syringe types should be tested in order to identify the brand and type allowing the most accurate and reproducible dosage. This recommendation should be considered when setting up minimum reporting standards for *in vivo* nanobiosciences as e.g. by MIRIBEL.

It should be the task of the manufacturers of syringes and medical injection equipment to identify the physicochemical reasons of this effect and to develop and to provide devices which are fit for purpose and avoid the necessity of cumbersome and time consuming additional checks. In the meantime the present authors believe that the present surprising and disturbing results require the suggested checks as good laboratory practice in order to make experimental designs more robust and improve the reproducibility of *in vivo* nanobiosciences studies.

## Data Availability Statement

The present article contains all available data at JRC and CIC biomaGUNE on nanoparticle retention in syringes. The raw data, i.e., radioactivities measured in syringes after loading and unloading, will be made available by the authors, without undue reservation.

## Author Contributions

WK and UH planned the syringe retention experiments. UH performed the syringe retention experiments and the radiation measurements at the JRC. UC and JL retrieved and re-evaluated syringe retention data from *in vivo* experiments with radiolabeled nanoparticles at CIC biomaGUNE. All authors contributed to the drafting of the manuscript.

## Funding

No funding was received for the present research.

## Conflict of Interest

WK was employed by Helmholtz Zentrum München which is a non-profit research institution of the Federal Republic of Germany and the Free State of Bavaria and a member of the Helmholtz Association of German Research Centers.

The remaining authors declare that the research was conducted in the absence of any commercial or financial relationships that could be construed as a potential conflict of interest.
